# Imaging Findings and Misdiagnosis of Bronchogenic Cysts: A Study of 83 Cases

**DOI:** 10.5334/jbsr.3214

**Published:** 2023-10-18

**Authors:** Xiaoyu Gu, Li Zhu, Yingming Li, Bo Yin, Zhongqiu Wang

**Affiliations:** 1 Department of Radiology, Affiliated Hospital of Nanjing University of Chinese Medicine, Nanjing 210009, China; 2 Department of Radiology, Huashan Hospital, Fudan University, Shanghai 200040, China; 3 Department of Medical Imaging, Yantai Yuhuangding Hospital, Yantai 264000, China

**Keywords:** bronchogenic cyst, computed tomography, magnetic resonance imaging

## Abstract

**Objective:**

We characterize computed tomography (CT) and magnetic resonance imaging (MRI) features of bronchogenic cysts (BCs) and analyze misdiagnosis.

**Methods:**

The retrospective study consisted of 83 patients with BCs. CT and MRI images were assessed for mass location, maximum diameter, density, calcification, signal intensity, and enhancement pattern. Eighty-three patients underwent *plain* CT in which 53 underwent *enhanced* CT. Thirteen patients received both plain and enhanced MR, and only one received just a plain MR.

**Results:**

Eighty-three masses were all solitary, with 71 having a roundish morphology, and twelve having a lobulated or irregular morphology. Sixty-six masses are mediastinal type, four are intrapulmonary type, and 13 are ectopic type. Calcification occurred in 14 lesions. On plain CT, 13 lesions displayed water-like attenuation (–20–20 Hu), and 70 showed soft-tissue attenuation (≥21 Hu). On T1WI, eight masses were hyperintense, three were isointense, and three were hypointense. Fourteen masses were hyperintense on T2WI and (Apparent Diffusion Coefficient) ADC sequence. On (Diffusion Weighted Imaging) DWI, six masses were hypointense and eight were hyperintense. Enhanced T1WI showed seven cases were unenhanced, while six were marginally enhanced. Twenty cases were misdiagnosed as thymomas, eleven as neurogenic tumors, six as lymphangiomas, and two as lung cancer. Five cases were misdiagnosed as other diseases. Patients with BCs underwent MR (42.9%) had a lower rate of misdiagnosis than those who underwent CT alone (53.0%).

**Conclusion:**

The imaging findings of BCs in the chest are generally consistent. Misdiagnosis occurs frequently when CT attenuation values exceed 20 Hu. Diagnostic accuracy of BCs tends to improve with preoperative MR examination.

## Introduction

Bronchogenic cysts (BCs) are benign malformations because of congenital abnormalities. There are three types of BCs: mediastinal, intrapulmonary, and ectopic, with the mediastinal type dominating [1]. Low incidence, uncertain components, and rare locations may easily lead to preoperative misdiagnosis [2]. Occasionally, BCs may undergo malignant transformation [3,4]. Hence, preoperative diagnosis of BCs is essential to the treatment and prognosis. Plain computed tomography (CT) scans are the primary imaging technique for BCs. Magnetic resonance imaging (MRI) displays lesion components better without radiation, improving imaging diagnostic accuracy. Symptomatic BCs need completely resected to prevent complications, malignant transformation, and recurrence. Whether or not to resect asymptomatic BCs depends on the patient’s age, the location, and the volume of the mass. Clinical and imaging data of 83 patients with pathologically, confirmed BCs were collected retrospectively. CT and MRI findings of BCs were summarized for misdiagnosis analysis.

## 1 Methods and Materials

### 1.1 Patient characteristics

**Figure 1:**
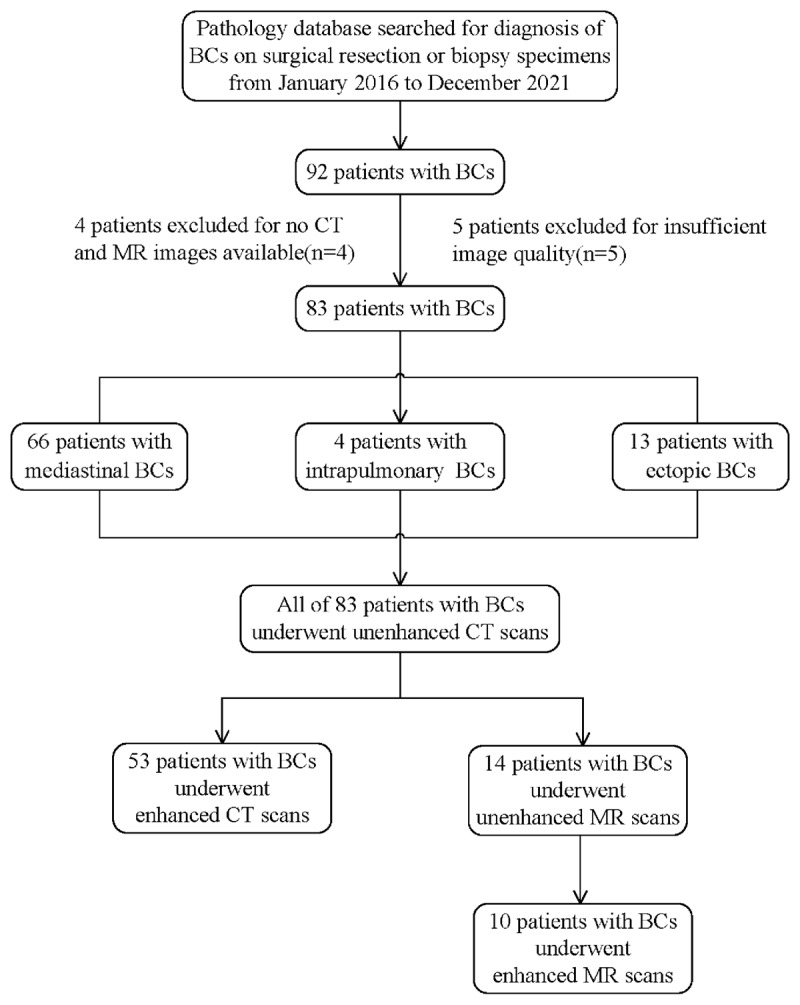
Study workflow of patient selection.

The surgical pathology database from one hospital was searched between January 2016 and December 2021 for patients with BCs. This retrospective study was approved with informed consent waived by the institutional review board. The exclusion and inclusion criteria (Figure 1) determined our study enrolled 83 patients with BCs.

### 1.2 Clinical information

The age of patients with BCs was 47.8±13.8 years (range, 11–76 years). The enrolled patients consisted of 39 males and 44 females. Clinical symptoms were present in 23 patients (Table 1). There were 44 cases of misdiagnosis prior to surgery (Table 4).

**Table 1:** Patient characteristics in the three groups of BCs.


CHARACTERISTICS	MEDIASTINAL	INTRAPULMONARY	ECTOPIC	*P* VALUE

Gender				0.086

Male	27(40.9%)	3(75.0%)	9(69.2%)	

Female	39(59.1%)	1(25.0%)	4(30.8%)	

Age	47.8±1.6	58.3±4.9	43.5±3.9	0.089

Clinical symptom				0.199

Absence	50(75.8%)	2(50.0%)	8(61.5%)	

Presence	16(24.2%)	2(50.0%)	5(38.5%)	

Swallowing difficulty	4	0	0	

Acid reflux	1	0	1	

Chest tightness	4	0	0	

Chest pain	2	0	0	

Coughing and expectoration	4	2	1	

Sore throat	1	0	1	

Subcutaneous lump	0	0	1	

Numbness in lower limbs	0	0	1	


### 1.3 CT and MRI examination

CT examinations were conducted on a variety of CT scanners (Light Speed VCT, GE Healthcare, USA; Brilliance 64, Philips Medical Systems, Netherlands; and Somatom Definition, Siemens AG, Medical Solutions, Germany). Parameters of CT are as follows: the detector collimation was 64 × 0.5–0.625 mm, the gantry rotation time was 0.4–0.5s, the tube voltage was 120 kVp, the reference tube current was 160~250 mAs, and the field view was 35–40 cm. Reconstruction of all images was accomplished from enhanced CT consisting of 0.75 mm slices with 0.5–5 mm reconstruction increments. To initiate enhanced CT, a continuous bolus of 80–100 ml of iopromide (300 mg/ml, Schering, Germany) was injected into an antecubital vein through an 18-gauge catheter, with 40 ml of saline solution (injection rate of 5 ml/s). Every enhanced CT was performed with a speed of 30 seconds for arterial phase, 60 seconds for portal phase, and 120 seconds for delayed phase. In average, the CTDIvol was 8–10 mGy, and DLP was 300–450 mGy·cm.

MR examinations were conducted with a 3.0 T scanner (Magnetom Verio, Siemens, Germany). Images were acquired using axial spin-echo T1WI (TR/TE, 500600/1420), axial fast spin-echo T2WI (TR/TE, 25004500/90110), and axial or coronal T2WI fluid-attenuated inversion recovery sequence (T2 FLAIR) (TR/TE/TI, 8000/130/2200). Certain patients were provided with diffusion-weighted images (single-shot spin-echo echoplanar sequence with b factors of 0 and 1,000 sec/mm^2^) in the axial plane. Axial turbo-FLASH T1WI images were obtained following intravenous injection of 0.1 mmol/kg gadolinium (3 ml/s, Leverkusen, Germany).

### 1.4 Pathology examination

Two experienced pathologists blindly interpreted pathological specimens using hematoxylin and eosin (HE) staining. The pathological criteria for BCs refer to pseudostratified ciliated columnar epithelium, along with smooth muscle cells, seromucous glands, or cartilage. Some characteristics may be distorted or destroyed by infection or malignancy.

### 1.5 Imaging and statistical analysis

A consensus interpretation was reached by two experienced radiologists blinded to pathology results. Imaging parameters included mass location, maximum diameter, calcification, attenuation values, and enhancement pattern. On plain CT, the mass density was classified as water-like attenuation (–20–20 Hu), or soft-tissue attenuation (≥21 Hu) [1]. Cystic and calcification components, if present, were avoided. Enhancement pattern was derived from the mass attenuation values of multiphasic CT. In the absolute CT attenuation values, an increase of 10–30 Hu is considered mild enhancement, 31–50 Hu as moderate enhancement, and ≥51 Hu as apparent enhancement. We categorized mass signal intensity according to its relative intensity to skeletal muscle, as hypointense, isointense, slightly hyperintense, and hyperintense. Mild, Moderate, and apparent enhancements were defined on MR.

SPSS26.0 was used as the statistical software. Data are presented as mean±SD, and count data as percentages. Quantitative data were compared using the Shapiro-Wilk (n ≤ 50) normality test. ANOVA (normal distribution) or Kruskal-Wallis Test (non-normal distribution) is applied to evaluate the groups. Chi-square test or Fisher’s exact test is applied to compare qualitative data. *P* < 0.05 is determined to be statistically significant.

## 2 Results

### 2.1 Clinical characteristics

**Figure 2:**
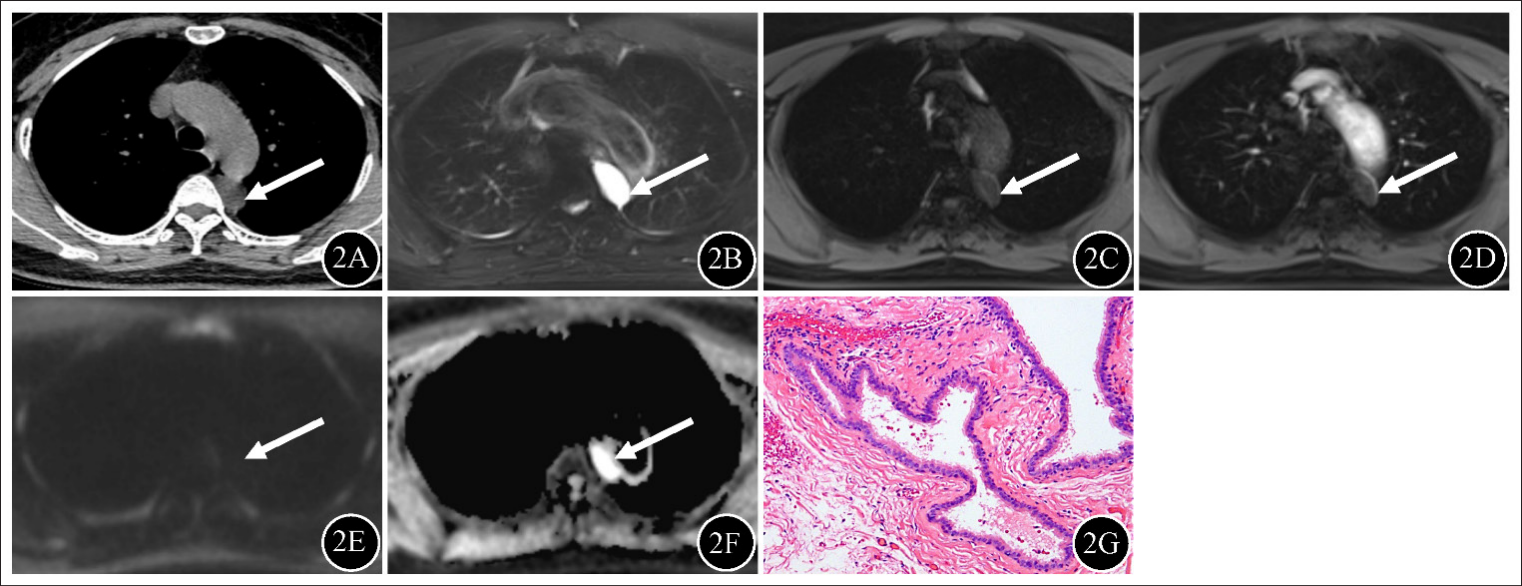
Posterior mediastinum BC (arrow) in a 53-year-old female. **2A** On unenhanced CT, a hypodense mass was observed in the left posterior mediastinum, with attenuation values of 12.0 Hu. **2B** On fat-suppressed T2WI, the mass showed homogeneous hyperintensity. **2C–D** On T1WI, the mass appeared hypointense with marginal enhancement. **2E** The signal intensity of the mass on DWI was hypointense. **2F** The ADC values for the mass were 2.61 × 10^–3^ mm^2^/sec. 2G Photomicrograph showed typical pseudostratified ciliated columnar epithelium structure (hematoxylin and eosin staining; original magnification, ×100).

**Figure 3:**
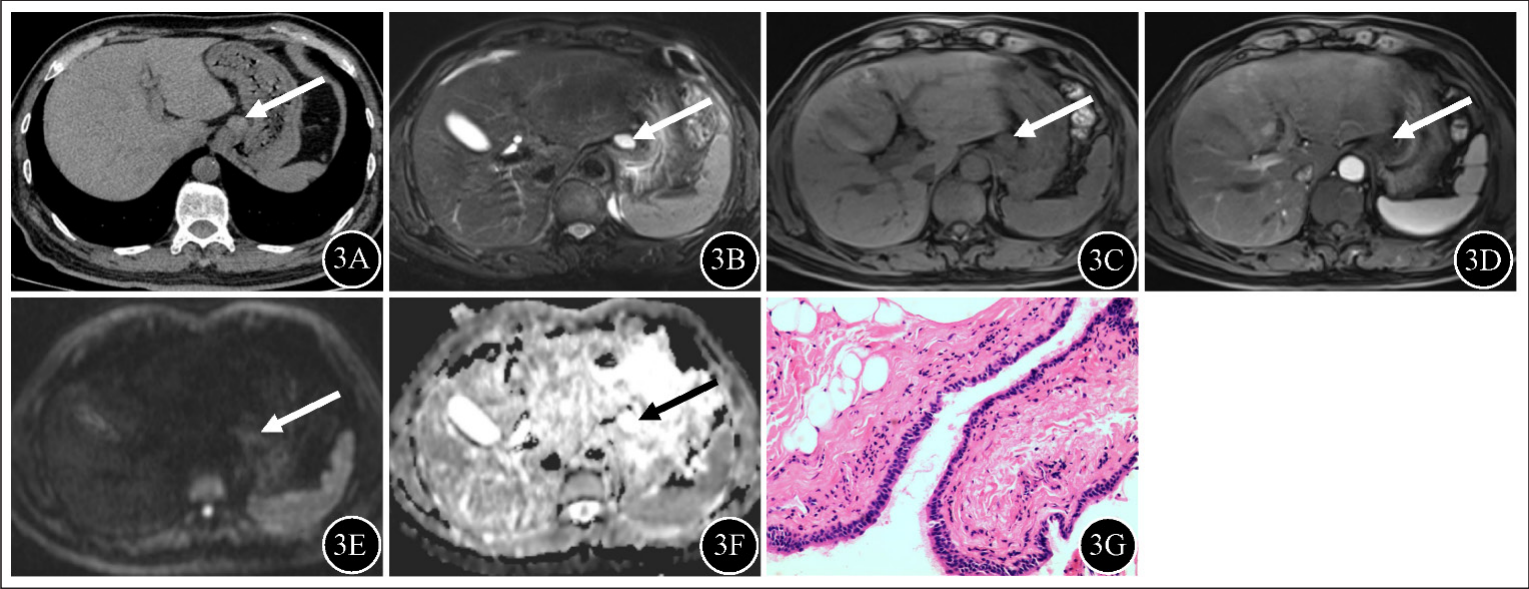
Gastric cardia BC (arrow) in a 67-year-old male. **3A** Unenhanced CT revealed a soft-tissue attenuation mass in the gastric cardia, with attenuation values of 45.9 Hu. **3B** The mass was homogeneous hyperintense on fat-suppressed T2WI. **3C–D** T1WI revealed that the mass was hypointense without enhancement. **3E** The signal intensity of the mass on DWI was hyperintense. **3F** The ADC values for the mass were 2.83 × 10^3^ mm^2^/sec. **3G** Typical pseudostratified ciliated columnar epithelium structure was found on photomicrograph (hematoxylin and eosin staining; original magnification, ×100).

**Figure 4:**
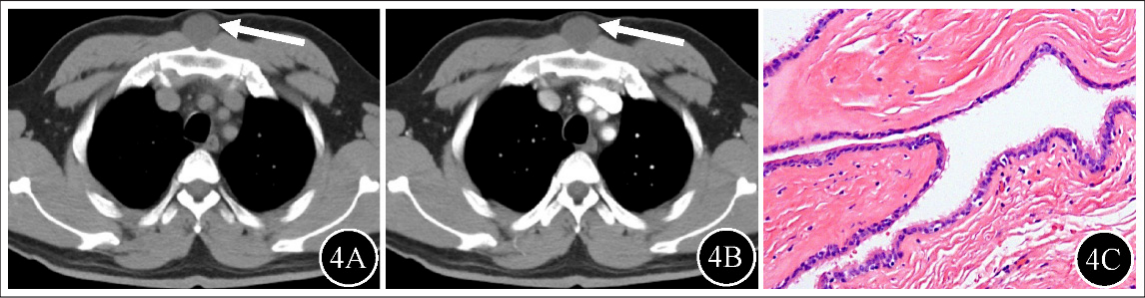
Chest wall BC (arrow) in a 30-year-old male. **4A** Unenhanced CT showed a hypodense mass in the chest wall, with attenuation values of 21.1 Hu. **4B** There was no enhancement of the mass on enhanced CT. **4C** Photomicrograph demonstrated typical pseudostratified ciliated columnar epithelium structure (hematoxylin and eosin staining; original magnification, ×100).

Patient characteristics are summarized in Table 1. 66 masses are mediastinal type (Figure 2, posterior mediastinal BC), four are intrapulmonary type, and 13 are ectopic type (Figure 3, gastric cardia BC; Figure 4, chest wall BC). Males constituted the majority of mediastinal BCs, whereas females constituted the majority of intrapulmonary and ectopic BCs, with a statistically significant difference between the groups (*P* < 0.05). Clinical symptoms did not differ significantly between the groups (*P* > 0.05).

### 2.2 CT and MR imaging findings

CT and MR imaging findings of BCs are summarized in Table 2. There was no significant difference in the maximum diameter between the groups (*P* > 0.05). Calcification occurs in 14 of 83 cases (16.9%). Most lesions retained a roundish morphology. Calculation and morphology did not differ statistically among the three groups (*P* > 0.05). No statistical difference was found in plain CT attenuation values between the groups (*P* > 0.05). Of the 53 cases that underwent enhanced CT, 47 were without enhancement, and six were slightly marginally enhanced.

**Table 2:** CT and MR imaging findings in the three groups of BCs.


CHARACTERISTICS	MEDIASTINAL	INTRAPULMONARY	ECTOPIC	*P* VALUE

Maximum diameter(cm)	2.7 ± 0.2	3.0 ± 0.7	2.9 ± 0.4	0.612

Calcification				0.332

Presence	9(13.6%)	1(25.0%)	4(30.8%)	

Absence	57(86.4%)	3(75.0%)	9(69.2%)	

Morphology				0.209

Roundish	58(87.9%)	2(50.0%)	11(84.6%)	

Lobulated/irregular	8(12.1%)	2(50.0%)	2(15.4%)	

Plain CT attenuation(Hu)	36.3 ± 1.9	29.7 ± 7.9	37.5 ± 4.6	0.652

Water-like attenuation	10(15.1%)	2(50.0%)	1(7.7%)	

Soft-tissue attenuation	56(84.9%)	2(50.0%)	12(92.3%)	

CT C+	40	4	9	-

Unenhanced	35(87.5%)	3(75.0%)	9(100.0%)	

Slightly marginal enhanced	5(12.5%)	1(25.0%)	0(0.0%)	

T1WI	11	0	3	-

Hypointense	2(18.2%)	0(0.0%)	1(33.3%)	

Isointense	3(27.3%)	0(0.0%)	0(0.0%)	

Hyperintense	6(54.5%)	0(0.0%)	2(66.7%)	

T2WI	11	0	3	-

Hypo/isointense	0(0.0%)	0(0.0%)	0(0.0%)	

Hyperintense	11(100.0%)	0(0.0%)	3(100.0%)	

DWI	11	0	3	-

Hypointense	6(54.5%)	0(0.0%)	0(0.0%)	

Hyperintense	5(45.5%)	0(0.0%)	3(100.0%)	

ADC	11	0	3	-

Hypo/isointense	0(0.0%)	0(0.0%)	0(0.0%)	

Hyperintense	11(100.0%)	0(0.0%)	3(100.0%)	

T1WI C+	10	0	3	-

Unenhanced	6(60.0%)	0(0.0%)	1(33.3%)	

Slightly marginal enhanced	4(40.0%)	0(0.0%)	2(66.7%)	


Fourteen patients received unenhanced MR, 13 of whom received enhanced T1WI. The signal intensity on T1WI, T2WI, DWI, ADC sequence, and enhanced T1WI was revealed in Table 2. Seven cases without enhancement, and six with slightly marginal enhancement were observed on enhanced T1WI.

### 2.3 Diagnosis and misdiagnosis results

**Figure 5:**
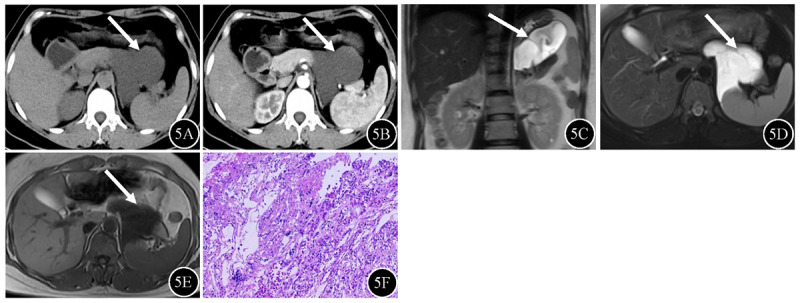
Cystic lymphangioma (arrow) in a 43-year-old female. **5A** Unenhanced CT revealed a water-like attenuation mass between the stomach and pancreas, with attenuation values of 16.8 Hu. **5B** Enhanced CT showed the mass was hypodense without enhancement. **5C–D** The mass was hyperintense on coronal T2WI and axial fat-suppressed T2WI, with multilocular characteristics. **5E** The signal intensity of the mass on T1WI was hypointense. **5F** Flat endothelial cells, loose connective tissue and scattered lymphocytes were observed on photomicrograph (hematoxylin and eosin staining; original magnification, ×100).

**Figure 6:**
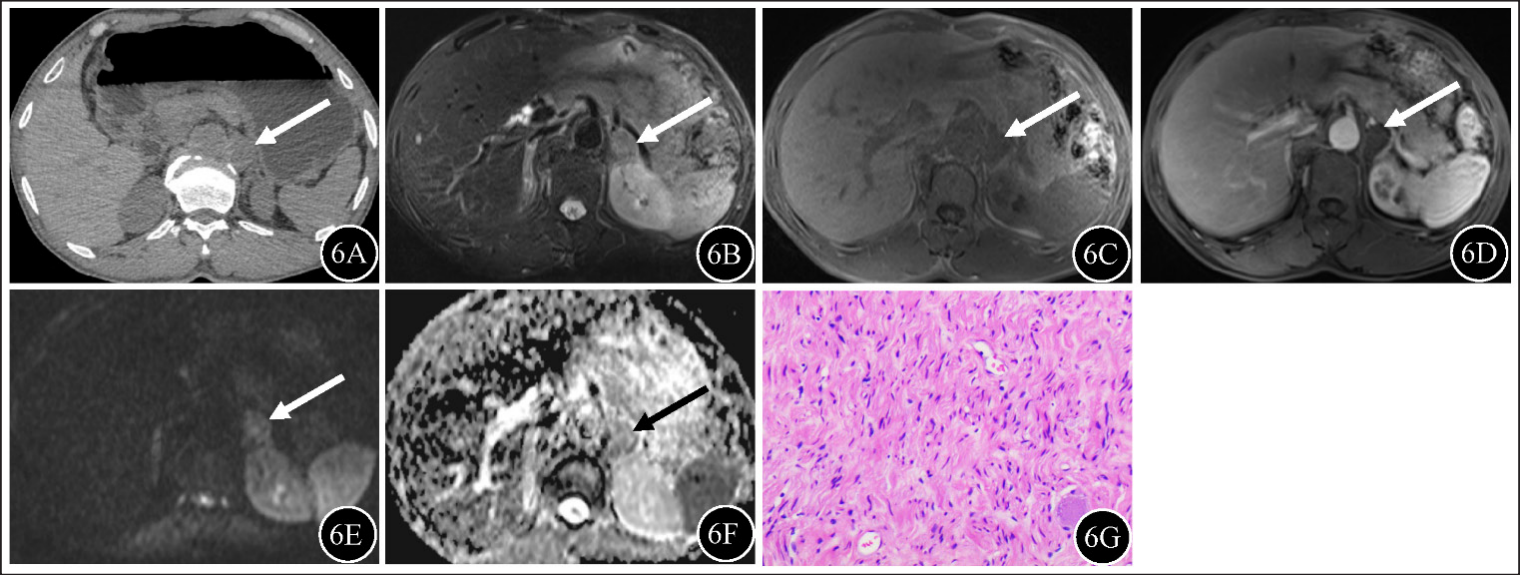
Ganglioneuroma (arrow) in a 49-year-old male. **6A** Unenhanced CT revealed a soft-tissue attenuation mass on the left side of the abdominal aorta, with attenuation values of 31.7 Hu. **6B** The mass was slightly hyperintense on fat-suppressed T2WI. **6C–D** T1WI revealed that the mass was hypointense without enhancement. **6E** The signal intensity of the mass on DWI was hyperintense. **6F** The ADC values for the mass were 1.71 × 10^–3^ mm^2^/sec. **6G** Disorganized spindle cells were found on photomicrograph (hematoxylin and eosin staining; original magnification, ×100).

A comparison of patient characteristics and imaging findings between the confirmed group and the misdiagnosis group is presented in Table 3. Diagnosis and misdiagnosis results are shown in Table 4. Preoperative imaging diagnosis was consistent with pathological findings in 13 cases, and 44 cases were misdiagnosed. Patients with BCs who only underwent CT had a misdiagnosis rate of 53.0% (44/83), while those who underwent MR had a misdiagnosis rate of 42.9% (6/14). CT attenuation values of BCs are mostly greater than 20 Hu, but lack of enhancement and hyperintensity on T2WI indicate benign characteristics. Rare locations, such as the chest wall, gastrointestinal tract, and retroperitoneum, or hyperintense on T1WI, may lead to misdiagnosis of BCs. One case of cystic lymphangioma and one case of ganglioneuroma were presented in Figures 5 and 6.

**Table 3:** Patient characteristics and imaging findings of confirmed group and misdiagnosis group.


CHARACTERISTICS	CONFIRMED GROUP	MISDIAGNOSIS GROUP	*P* VALUE

Gender			0.559

Male	17(43.6%)	22(50.0%)	

Female	22(56.4%)	22(50.0%)	

Age	46.0±2.3	49.4±2.0	0.587

Clinical symptom			0.071

Absence	24(61.5%)	22(50.0%)	

Presence	15(38.5%)	22(50.0%)	

Maximum diameter(cm)	3.3±0.3	2.3±0.1	0.005

Calcification			0.804

Presence	7(17.9%)	7(15.9%)	

Absence	32(82.1%)	37(84.1%)	

Morphology			0.821

Roundish	33(84.6%)	38(86.4%)	

Lobulated/irregular	6(15.4%)	6(13.6%)	

Plain CT attenuation(Hu)	32.7±2.4	39.2±2.2	0.045

CT C+	26	27	-

Unenhanced	26(100.0%)	21(77.8%)	

Slightly marginal enhanced	0(0.0%)	6(22.2%)	

T1WI	9	5	-

Hypointense	2(22.2%)	1(20.0%)	

Isointense	3(33.3%)	0(0.0%)	

Hyperintense	4(44.5%)	4(80.0%)	

T2WI	9	5	-

Hypo/isointense	0(0.0%)	0(0.0%)	

Hyperintense	9(100.0%)	5(0.0%)	

DWI	9	5	-

Hypointense	5(55.6%)	1(20.0%)	

Hyperintense	4(44.4%)	4(80.0%)	

ADC	9	5	-

Hypo/isointense	0(0.0%)	0(0.0%)	

Hyperintense	9(100.0%)	5(0.0%)	

T1WI C+	8	5	-

Unenhanced	6(75.0%)	1(20.0%)	

Slightly marginally enhanced	2(25.0%)	4(80.0%)	


**Table 4:** Diagnosis and misdiagnosis results in the three groups of BCs.


CHARACTERISTICS	MEDIASTINAL	INTRAPULMONARY	ECTOPIC	*P* VALUE

Confirmed group	36	1	2	0.001

Bronchogenic cyst	12(33.3%)	1(100.0%)	0(0.0%)	

Mediastinal cyst	24(66.7%)	0(0.0%)	0(0.0%)	

Dermoid cyst	0(0.0%)	0(0.0%)	2(100.0%)	

Misdiagnosis group	30	3	11	<0.001

Thymoma	20(66.7%)	0(0.0%)	0(0.0%)	

Nasal polyp	0(0.0%)	0(0.0%)	1(9.1%)	

Neurogenic tumor	9(30.0%)	0(0.0%)	2(18.2%)	

Lymphangioma	1(3.3%)	0(0.0%)	5(45.4%)	

Lung cancer	0(0.0%)	2(66.7%)	0(0.0%)	

Tuberculosis	0(0.0%)	1(33.3%)	0(0.0%)	

Gastrointestinal stromal tumor	0(0.0%)	0(0.0%)	1(9.1%)	

Adrenal adenoma	0(0.0%)	0(0.0%)	1(9.1%)	

Teratoma	0(0.0%)	0(0.0%)	1(9.1%)	


## 3 Discussion

BCs are congenital benign abnormalities of the primitive foregut during embryogenesis, resulting in abnormal budding separating from the normal tracheobronchial tree [5]. Most BCs occur in the mediastinum, followed by the chest, and they are rarely present in the retroperitoneum and intracalvarium [6,7]. Our study found only four cases of intrapulmonary BCs, probably because some lesions contained gas and improved with conservative treatment rather than surgical resection.

During this study, 60 (72.3%) asymptomatic patients with BCs were found following a routine physical examination, and 23 (27.7%) symptomatic patients with BCs were identified. Patients may experience clinical symptoms such as fever, cough, or pain because of bleeding from the masses, or secondary infection. McAdam et al. reported BCs generally distributed along the midline of the body with roundish appearances [1]. Increased secretions can enlarge the mass volume, compress nearby organs, and cause associated clinical symptoms [8,9].

On CT, 13 (15.7%) masses were water-like attenuation, and 70 (84.3%) masses were soft-tissue attenuation in our study. Jeon et al. reported that if the cysts are not infected and consist of few protein components, they will display water-like attenuation with thin fluid; however, infected cysts composed of a large quantity of protein components may present with soft-tissue attenuation and marginal enhancement. Intracapsular bleeding can also increase the attenuation values of BCs [10]. The characteristic CT features of mediastinal BCs are water-like attenuation values without enhancement. We found calcification of the cyst wall in 14 of 83 cases. A 16.4% rate of calcification was observed, slightly higher than the 10% reported by McAdam et al. [1].

MR has the advantage of showing cystic components that outweigh CT. T1WI will show hyperintense mass if the protein components exceed the fiber components or if intracapsular bleeding occurs. It is likely that the mass on T1WI will be isointense if the protein components are approximately equal to the fiber components. The fewer the protein contents are, the lower the signal intensity of mass on T1WI is. On T1WI, we recorded eight hyperintense, three isointense, and three hypointense masses. Generally, hyperintense masses on T2WI indicate cystic contents; in combination with apparent high ADC values, hyperintense masses on DWI may indicate benign characteristics [11]. On enhanced MR, the mass can be marginal enhanced when infected. The characteristic MR features of mediastinal BCs include hypointense on T1WI without enhancement, hyperintense on T2WI, hypointense on DWI, and high ADC values.

The main reasons for misdiagnosis of BCs are as follows: the scanning is mostly demonstrated soft-tissue attenuation on unenhanced CT, which barely reflects blood supply and cystic contents (infected lesions can be slightly enhanced). Hyperintense on T1WI interferes with the radiologist’s observations of the mass enhancement pattern. T1 subtraction technique can eliminate the original hyperintense on T1WI, which enhances the accuracy of determining the enhancement pattern.

Mediastinal BCs should be distinguished from thymomas and neurogenic tumors. The solid components of thymomas tend to be enhanced [12]. When evaluating mediastinal BCs, MRI is more effective than CT, as the cystic components are usually hypointense or slightly hyperintense on T1WI, while appearing hyperintense on T2WI. A lack of enhancement can also be found on subtraction imaging for mediastinal BCs [13]. There should be a strong suspicion of thymoma whenever an anterior mediastinal mass is associated with symptoms of myasthenia gravis or other related paraneoplastic syndrome [14]. It is more likely to be BCs due to the thin cystic wall and movement during the inspiratory and expiratory phases on CT. BCs may reveal fluid-fluid level on fat-suppressed T2WI and without enhancement on T1WI. Neurogenic tumors, such as schwannomas and ganglioneuromas, usually have homogeneous soft-tissue attenuation on CT without enhancement and show partial enhancement of the parenchyma despite the necrotic cystic region exists. Schwannomas may appear as the fascicular sign on fat-suppressed T2WI when peripheral high signal intensity is observed. The sympathetic chain determines the direction in which ganglioneuromas grow. Ganglioneuromas possess the target sign of peripheral hyperintense areas in conjunction with hypointense areas in the central portion of the mass on fat-suppressed T2WI, which corresponds pathologically to fibrous tissue centrally and myxoid tissue peripherally [15].

Intrapulmonary BCs can present non-specific clinical manifestations such as cough and sputum. Asymptomatic small lesions are often misdiagnosed and need to be distinguished from lung abscesses. Pulmonary abscesses are characterized by rapid progression, purulent sputum, and thick wall with obvious enhancement [16,17]. The clinical course of BCs is relatively slow, and the sputum is thin without enhancement. Ectopic BCs should be distinguished from teratomas and lymphangiomas. Teratomas are generally composed of bone, fat, and hair. Fat attenuation on CT and hypointensity on fat-suppressed T2WI help differentiate teratomas from BCs [18]. Cystic lymphangiomas are often secondary to traumas and inflammations, presenting as unilocular or multilocular thin-walled masses having water-like attenuation without enhancement [19]. Without intervention, it may rapidly enlarge, compressing adjacent tissues and organs, causing nausea, relative pain, and vomiting [20]. BCs, as congenital unilocular roundish anomalies without enhancement, can be observed as calcification of the cystic wall. Additionally, BCs grow slowly in volume.

The present study had limitations and deficiencies. Firstly, there were only 14 patients who received MR scans. Secondly, the number of intrapulmonary BCs was low. Hence, the varied imaging features of BCs need further summary and analysis.

## Conclusion

The imaging findings of BCs in the chest are generally consistent. Misdiagnosis occurs frequently when CT attenuation values exceed 20 Hu. Diagnostic accuracy of BCs tends to improve with preoperative MR examination.

## Data Accessibility statement

All relevant data are within the paper after the reference list.
